# Implementation of pre-clinical methodologies to study fibrosis and test anti-fibrotic therapy

**DOI:** 10.1016/j.coph.2019.10.004

**Published:** 2019-12

**Authors:** Fiona Oakley, Lucy M Gee, Neil S Sheerin, Lee A Borthwick

**Affiliations:** 1 Newcastle Fibrosis Research Group, Institute of Cellular Medicine, Newcastle University, Newcastle upon Tyne, UK; 2 Renal Department, Freeman Hospital, Newcastle upon Tyne, UK; 3 Applied Immunobiology and Transplantation Research Group, Institute of Cellular Medicine, Medical School, Newcastle University, Newcastle upon Tyne, UK

## Abstract

Diseases where fibrosis plays a major role accounts for enormous morbidity and mortality and yet we have very little in our therapeutic arsenal despite decades of research and clinical trials. Our understanding of fibrosis biology is primarily built on data generated in conventional mono-culture or co-culture systems and *in vivo* model systems. While these approaches have undoubtedly enhanced our understanding of basic mechanisms, they have repeatedly failed to translate to clinical benefit. Recently, there had been a push to generate more physiologically relevant platforms to study fibrosis and identify new therapeutic targets. Here we review the state-of-the-art regarding the development and application of 3D complex cultures, bio-printing and precision cut slices to study pulmonary, hepatic and renal fibrosis.

**Current Opinion in Pharmacology** 2019, **49**:95–101

This review comes from a themed issue on **Fibrosis**

Edited by **Lee Borthwick** and **Fiona Oakley**

For a complete overview see the Issue and the Editorial

Available online 12th November 2019

**https://doi.org/10.1016/j.coph.2019.10.004**

1471-4892/© 2019 The Authors. Published by Elsevier Ltd. This is an open access article under the CC BY license (http://creativecommons.org/licenses/by/4.0/).

## Introduction

Fibrosis is the replacement of functional tissue architecture with excess fibrous connective tissue, leading to a reduction in organ function and ultimately organ failure and death. Fibrosis can affect all tissues in the body and therefore is a ubiquitous problem that contributes massively to morbidity and mortality worldwide [1]. While fibrosis is the common end-point for a wide range of diseases, the underlying aetiologies and mechanisms can be either core or organ specific, and in the majority of cases remain ill-defined/idiopathic [2]. There are only two approved anti-fibrotic therapies (Pirfenidone and Nintedanib) and both are licenced exclusively for the treatment of patients with mild-moderate Idiopathic Pulmonary Fibrosis (IPF) [3]. There is therefore an urgent unmet need to develop new anti-fibrotic therapies for use in other fibrotic diseases.

The global burden of fibrosis and lack of treatment options has led to the development of a wealth of experimental approaches to illuminate the underlying cellular and molecular mechanisms driving fibrosis, with the goal to identify new therapeutic targets [4]. The most commonly utilised model systems use human or rodent cells (both immortalised cell lines and primary cells) in conventional 2D submerged mono-cultures or co-cultures exposed to exogenous stimuli (e.g. Transforming Growth Factor-β1 (TGF-β1), matrix stiffness) to drive fibrogenesis/fibrosis *in vitro* [5]. Routinely these *in vitro* models are complemented by *in vivo* animal models of fibrosis, in a range of species from invertebrates to large mammals, with the most commonly employed experimental system being mouse models. While these models have undoubtedly provided valuable insights into our understanding of fibroblast biology and aspects of disease progression, these data have frequently failed to yield the necessary clinical benefit. Currently the probability of a drug progressing from Phase I to approval is <10% despite large investments in drug development [6,7]. One prominent explanation is flawed preclinical research, in which the use and outcome of animal models or non-physiological human systems is used to bridge the translational gap to the clinic.

**Figure 1:**
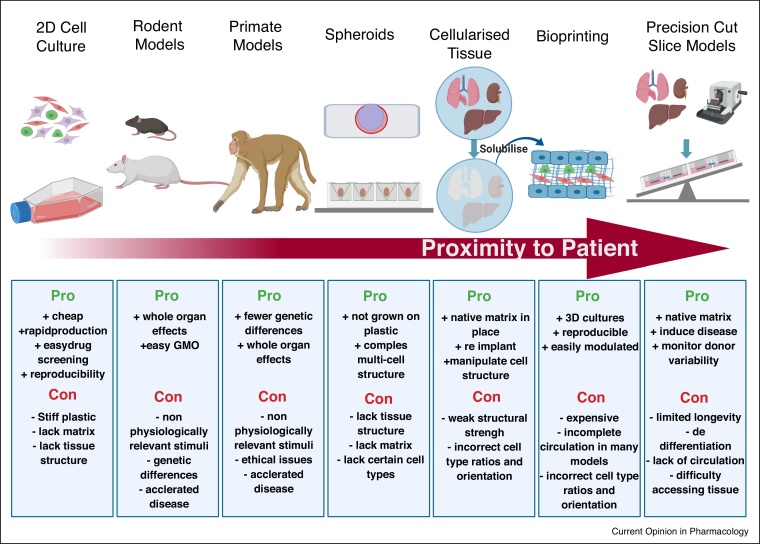
Models of organ fibrosis. A summary of the seven main research methodologies used to model fibrotic disease, along with the main advantages and disadvantages of each method. The breadth of models are ranked by the proximity of the model to the patient disease. Created with BioRender.com.

Fibrosis is a tightly regulated and dynamic process that involves a wide range of cell types, numerous cytokines/chemokines/growth factors and multiple cell–cell and cell–matrix interactions that drive concurrent biological processes in the complex microenvironment of human tissue. Widely utilised animal models and conventional 2D mono-culture and co-culture systems [8,9] fail to recreate the complex interactions seen in human tissue and thus mechanisms driving fibrosis need to be interrogated in more representative, complex human tissue systems. In this review, we will describe the state-of-the-art of the quest to develop more physiologically relevant *in vitro*/*ex vivo* cell culture systems to model fibrosis, focussing particularly on exciting recent advances in complex 3D cell culture models, bio-printing and precision cut slice (PCS) methodologies (Figure 1).

## Spheroids

Single or multicellular spheroids can be formed as hanging droplet cultures under gravity or in cell-repellent or ultra-low attachment plates. This methodology allows the rapid production of numerous spheres for studying disease biology, drug screening and toxicity studies.

To this end, hanging droplet microtissues comprising human hepatocyte, hepatic stellate cells (HSC) and Kupffer cell (KC) cell lines were engineered to model drug-induced fibrosis, whilst addition of lipopolysaccharide was used to evoke an inflammatory response [10]. Leite *et al.* created 3D liver spheroids from HepaRG cells and primary HSC to concurrently model fibrogenesis and hepatotoxicity. The organoids retained cell-specific markers, cytochrome p450 expression and albumin production for 21 days. Hepatotoxic compounds; allyl alcohol, paracetamol or methotrexate induced HSC activation, inflammation and ECM gene expression [11].

HSC rapidly transdifferentiate on plastic, whereas, HSC spheroid monocultures maintain a quiescent phenotype in culture until transferred to plastic where they rapidly activate. HSC spheroids were used to delay HSC activation and deliver siRNA’s targeting yes-associated protein (YAP), a transcriptional transducer of mechanical stress, to blunt HSC activation [12^•^]. This proof-of-concept study identifies a system where quiescent HSC can be modified, to identify proteins important for HSC activation.

## Tissue mimics

Nugraha *et al.* developed a 3D-dextran hydrogel model to artificially construct a renal tubule and model epithelial-myofibroblast crosstalk, under normal and disease-induced conditions. HCK-8 spheroids cultivated in hydrogel form tight junctions and acquire cellular polarity. Quiescent primary human myofibroblasts were then overlaid in a second gel layer, encapsulating the tubule structure. Nephrotoxin exposure promoted loss of E-cadherin, myofibroblast proliferation and induction of fibrosis genes [13^••^]. Pirfenidone blunted this response, highlighting the translational relevance of this model. Cell lines can be easily genetically modified to modulate protein expression or monitor biological responses in real-time. Here, HCK-8 cells expressed E-cadherin-GFP as a ‘biosensor’ of epithelial differentiation.

A 3D space of Disse mimic was created from primary rat hepatocytes cultured on collagen coated plates, separated from a heterogeneous layer of sinusoidal endothelial cells, HSC and KC by a detachable polyelectrolyte multilayer (PEM). Normal PEM or PEMs with increasing mechanical stiffness (21–43 kPa) were used to assess the influence of substrate elasticity on HSC activation in the context of physiological cell:cell crosstalk [14]. Akin to 2D and 3D cultures, increasing mechanical stiffness accelerated HSC activation and fibrogenic phenotype.

## Decellularised tissue

Decullularised or acellular tissue scaffolds from normal or fibrotic organs that are repopulated with single or multiple cell lines or primary cells have been developed over the past decade to study fibrosis and perform drug feasibility studies. Standardised perfusion and immersion methods to generate and manipulate decellularised tissue scaffolds, the clinical applications and limitations of the technology have been recently reviewed [15, 16, 17, 18].

For fibrosis, Booth *et al.* reported that the native tissue structure, stiffness and ECM composition is preserved in acellular tissue scaffolds created from normal or fibrotic human lung. Interestingly, lung fibroblasts cultured in normal scaffolds have low αSMA expression whereas myofibroblast markers are significantly increased when fibroblasts are cultured in fibrotic scaffolds, a response that is independent of TGFβ [6]. A follow-up study revealed that these acellular lung scaffolds populated with iPSC could be exploited to study fibrosis biology [19]. In liver, Pinzanni‘s group optimised a retrograde perfusion protocol to decellularise human liver tissue and fashion acellular cubes that retain the native ECM composition and structure [20]. Hepatocyte and HSC cell lines seeded into these scaffolds were preserved in culture and the cellularised scaffolds were successfully xenografted into immunocompetent mice.

He *et al.* recently described the optimal conditions to generate decellularised rat kidney to preserve structure, collagen, fibronectin, glycosaminoglycans and laminin. Interestingly, this group reseeded the kidney scaffold by directly injecting either primary renal cell or bone marrow derived mesenchymal stromal cells (MSCs) through the vasculature, and then maintained flow through the scaffold to aid the recellularization process. Histological analysis of the recellularized tissue revealed glomerular-like structures, which express both epithelial and endothelial markers, suggesting that micro-flow may facilitate the repopulation process [21].

Tissue stiffness and ECM composition can modulate myofibroblast behaviour [22]. To dissect the contribution of these environmental cues to pericytes-myofibroblast differentiation, human pericytes were grown on hydrogels permeated with soluble decellularised normal or fibrotic lung matrix, bioengineered to have tuneable mechanical properties. In short-term cultures, αSMA expression was significantly induced in pericytes cultured on IPF matrix compared to normal matrix, and this activation was supressed by Nintedanib. Matrix stiffness was the dominant regulator of pericyte activation, independent of matrix composition or TGFβ stimulation. However, prolonged exposure to ECM or fibrotic stimuli was sufficient to drive pericyte differentiation [23^••^].

## Bioprinting

Bioprinting allows rapid and highly reproducible automated fabrication of biomimetic tissue models. Cellular composition, environmental substrates and soluble factors collectively termed ‘bioinks’ can be customised to create bespoke 3D culture models with bioactive matrices. Fusion of bioprinting and bioengineering technologies provide an opportunity to manufacture ‘modulatable systems’ where the physical properties of ECM, for example, stiffness or crosslinking can be chemically or photo-activated/inactivated. For further details of Bioinks and bioprinting see Ref. [24].

A bioprinted human liver comprising cryopreserved primary human hepatocytes, HSC and umbilical vein endothelial cells was developed by Norona *et al.* to model drug or TGFβ induced tissue injury, inflammation and fibrosis [25]. In a follow-up study, KC were integrated into the model to evaluate parenchymal-macrophage-mesenchymal cell signalling during drug induced liver injury and fibrosis [26^••^]. Recently, Ma *et al.* bioprinted HepG2 cells suspended in decellularized matrix and used digital light processing to photo-crosslink the matrix scaffold to mimic mechanical stiffness in the normal (0.5 kPa) and diseased liver (15 kPa). Although this system has been developed to evaluate the role of matrix rigidity on liver cancer cell growth and migration, the technology could be modified to study fibrosis [27].

Whilst the field is in its infancy and application has mainly focused on regenerative medicine for many organs, the field is rapidly evolving. In the future, it could have great value in illuminating mechanisms of tissue fibrosis and for anti-fibrotic drug discovery.

## Precision cut slices

Complex 3D models are useful drug discovery tools but fail to fully recapitulate the cellular heterogeneity and physiological components of the native tissue. Precision cut slices (PCS) produced from either normal or diseased, rodent or human tissue, offer a more faithful model to study fibrosis [28, 29, 30]. Importantly, human PCS are a valuable tool to validate discoveries in other preclinical models and test emerging anti-fibrotic medicines in a clinically relevant system.

Normal mouse precision cut kidney slices (PCKS) cultured in 80% oxygen have increased inflammatory gene expression within 3 hours, whilst fibrosis gene expression was elevated by 96 hours. Treatment with LY2109761, a TGFβ receptor inhibitor, blunted the fibrogenic response confirming that spontaneous fibrosis was in part TGFβ-dependant [31]. Using this system, successful myofibroblast-specific targeting of an anti-fibrotic IFNγ-peptide conjugate (PPB-PEG-IFNγ) limited TGFβ-induced fibrogenic gene expression and collagen deposition [32], providing evidence that peptide therapies can penetrate tissue slices and exert biological effects.

To model interstitial kidney fibrosis, PCKS were generated from unilateral ureteral obstruction (UUO) rats. Nordic extracellular matrix neo-epitopes of collagen formation (P1NP) and collagen degradation (C1M and C3M) were significantly raised in the media of UUO PCKS compared to sham controls PCKS [33]. Active TGFβ was detectable in cultured fibrotic but not normal PCKS, suggesting establishment of a positive fibrogenic signalling feedback loop.

A feasibility study by Stribos *et al.* revealed that TGFβ stimulation induced fibrosis in normal human PCKS without compromising viability or metabolic activity [34]. The anti-fibrotic actions of Butaprost, a prostaglandin E2 receptor agonist, were shown in both mouse and human fibrotic PCKS. Butaprost did not affect PCKS metabolism or viability and drug engagement was confirmed by attenuation of TGFβ/SMAD signalling [35], corroborating a common mechanism of drug action between rodents and humans.

To study cellular changes driving interstitial lung disease (ILD), normal human PCLuS without ILD, were challenged with a cocktail of pro-fibrogenic and pro-inflammatory factors; TGFβ, TNFα, PDGFAB, and lysophosphatidic acid [36]. In culture, the fibrogenic cocktail promoted alveolar thickening, induced fibrosis and inflammatory gene expression and stimulated the secretion and deposition of ECM proteins, without effecting tissue viability or mitochondrial function. This methodology provides a useful tool system to interrogate the molecular and cellular mechanisms driving the pathogenesis of ILD/IPF.

Lehmann *et al.* used normal and disease murine primary alveolar epithelial type II (pmATII) and human PCLuS to identify the cellular targets of Pirfenidone and Nintedanib. Both drugs inhibited collagen and fibronectin expression in pmATII and PCLuS. However, only Nintedanib supressed changes in epithelial plasticity, suggesting that Nintedanib targets multiple cell types within the fibrogenic niche [37].

Fibrosis is both a progressive and reversible disease. Hansen *et al.* modelled ECM dynamics in rat fibrotic lung slices (fPCLuS) and reported that disease-relevant biomarkers; P1NP, C1M, P3NP,C3M and ELM7 (an elastin degradation marker) are increased in cultured fPCLuS but blunted by an MMP or phosphodiesterase inhibitor [38]. Anti-fibrotic actions of an ALK5 inhibitor and Nintedanib have been reported in fPCLuS created from chronic bleomycin injured mice, consistent with previous *in vitro* and *in vivo* studies [39]. Moreover, in agreement with observations in 2D human bronchial epithelial cultures, caffeine was shown to block epithelial activation of TGFB1, limiting fibrogenic gene expression and collagen production in fPCLuS [40].

Mechanistic studies in IPF fibroblasts cultured under 3D macromolecular conditions [41] and in human IPF PCLuS [42] revealed that matrix production was regulated by a TGF-β1-mTORC1-4E-BP1 dependant, PI3K independent, signalling axis. Proteomic studies on IPF PCLuS revealed that novel inhibitors of this pathway attenuated the secretion of matrisome factors and the biomarker P1NP.

TGF-β1/PDGF-β1 co-stimulation induces a robust fibrogenic response in normal PCLS without causing toxicity [43]. A number of anti-fibrotic compounds showing efficacy in 2D cultures or animal models have been validated in rodent and/or human precision cut liver slices (PCLS) [43, 44, 45]. Recently, Galunisertib, a TGF-β receptor type I kinase inhibitor, was shown to block TGFβ1 signalling, fibrogenic gene expression and ECM production in rodent and human PCLS [46]. Whilst targeting integrin alpha 11 (ITGA11) using Erismodegib reduced expression of fibrogenic genes and transcriptional targets of integrin-hedgehog signalling in hPCLS [47].

The PI3K/mTOR pathway is activated in multiple fibrotic diseases and is likely a common mechanism of fibrosis therefore testing pharmacological inhibitors of this pathway in multiple organs systems will have clinical value. The PI3K/mTOR inhibitor Omipalisib supressed AKT phosphorylation and fibrosis gene expression in normal or diseased (cholestatic mdr2^−/−^ mice or mice with NAFLD) PCLS. Omipalisib also reduced fibrogenic responses in normal and cirrhotic human PCLS. However, Omipalisib caused toxicity in cirrhotic human PCLS and intestinal slices [48], highlighting the importance of assessing drugs in advanced disease and investigating potential off target effects in other organs.

Typically, PCLS are cultured in high oxygen concentrations to offset the detrimental effects of tissue hypoxia. Recently, a bioreactor technology that creates flow through the PCLS was described to maintain functional PCLS — retaining all liver cell types and metabolic activity under normoxic conditions for six-days [49^••^]. Histological fibrosis, release of soluble ECM markers and expression of fibrogenic genes could be induced in normal rodent and human PCLS by co-stimulation with TGFβ1/PDGFβ1, a response that is attenuated by a number of antifibrotic drugs. Fibrogenesis was self-sustaining in bioreactor cultured rat fPCLS but limited by ALK5 inhibitor therapy. This bioreactor model was also used to demonstrate hepatoprotective and anti-fibrotic activities of the ammonia scavenger; ornithine phenylacetate in lipid-loaded and/or hyperammonaemia-treated human PCLS [50].

### Modelling different aetiologies of liver disease

A recent study demonstrated the feasibility of modelling the metabolic syndrome of liver disease by culturing rodent PCLS in supraphysiological levels of sugars, fat (palmitic acid) and insulin for 48 hours. Histological steatosis, elevated triglyceride levels and an increase in lipogenic gene expression were observed in disease-induced PCLS, consistent with pathology in patients with metabolic liver disease [51^••^]. The Chokshi group reported that PCLS treated with ethanol induced changes in mitochondrial morphology indicative of alcohol-induced hepatic injury, however, fibrosis was not investigated [29]. Whilst, Page *et al.* used ethanol stimulated rat PCLS to validate mechanistic studies showing that ethanol evokes epigenetic changes in HSC to regulate elastin production. Interestingly, PCLS treated with acetate, ethanol’s stable metabolite, failed to evoke the same response [52].

## Conclusion

Considerable research efforts have driven the development of more complex systems to study fibrosis mechanisms and test anti-fibrotic therapies. Current strategies to recreate disease-specific pathologies [29,50,51^••^,^52^] will complement the arsenal of existing research models, creating a breadth of disease models to test drug utility and efficacy. As complex 3D fibrosis models become more established, advanced molecular imaging for example, second-generation harmonics, RNA-sequencing or proteomic biomarker screens will become routine assays for anti-fibrotic drug discovery.

## Conflict of interest statement

LAB and FO are on the board of directors and hold shares in FibroFind Ltd.

## References and recommended reading

Papers of particular interest, published within the period of review, have been highlighted as:

• of special interest
•• of outstanding interest
